# Supplementary material for: Detailed molecular and epigenetic characterization of the pig IPEC-J2 and chicken SL-29 cell lines
Source: iScience. 2023 Feb 20;26(3):106252. doi: 10.1016/j.isci.2023.106252 (PMC10018572; doi:10.1016/j.isci.2023.106252)
Supplement: Data S2. Complete homer output for identified motifs in Chicken SL-29, related to Tables 5 and 6 — Homer motif analysis results for histone modifications H3K4me1, H3K4me3, H3K27ac, enhancers, and ATAC-seq of chicken SL-29 cell line. Parameters for possible false positives is as mentioned earlier for S5. [file mmc3.zip › Data_S2/S6/Chicken_SL_29/peak_files_enhancers/knownResults.html]

peak\_files\_enhancers - Homer Known Motif Enrichment Results


# Homer Known Motif Enrichment Results (peak\_files\_enhancers)

Homer *de novo* Motif Results  
Gene Ontology Enrichment Results  
Known Motif Enrichment Results (txt file)  
Total Target Sequences = 18518, Total Background Sequences = 31557

|  |  |  |  |  |  |  |  |  |  |  |  |
| --- | --- | --- | --- | --- | --- | --- | --- | --- | --- | --- | --- |
| Rank | Motif | Name | P-value | log P-pvalue | q-value (Benjamini) | # Target Sequences with Motif | % of Targets Sequences with Motif | # Background Sequences with Motif | % of Background Sequences with Motif | Motif File | SVG |
| 1 | C A T G C T A G T C G A A C G T A C T G C G T A T A G C C G A T T G A C C G T A A G C T G A T C | Fra2(bZIP)/Striatum-Fra2-ChIP-Seq(GSE43429)/Homer | 1e-604 | -1.391e+03 | 0.0000 | 2974.0 | 16.06% | 1700.9 | 5.39% | motif file (matrix) | svg |
| 2 | A C T G C T A G T C G A C G A T C A T G G C T A A T C G C G A T G T A C G C T A A G C T G T A C | Fra1(bZIP)/BT549-Fra1-ChIP-Seq(GSE46166)/Homer | 1e-569 | -1.312e+03 | 0.0000 | 3342.0 | 18.05% | 2147.8 | 6.81% | motif file (matrix) | svg |
| 3 | C T A G T C G A A C G T A C T G C G T A A T G C A C G T G T A C C G T A A G C T G A T C G T A C | Atf3(bZIP)/GBM-ATF3-ChIP-Seq(GSE33912)/Homer | 1e-568 | -1.308e+03 | 0.0000 | 3751.0 | 20.26% | 2596.8 | 8.23% | motif file (matrix) | svg |
| 4 | A C T G C A T G T C G A A C G T A C T G C G T A A T C G A C G T G T A C C G T A G A C T A G T C | Fos(bZIP)/TSC-Fos-ChIP-Seq(GSE110950)/Homer | 1e-554 | -1.277e+03 | 0.0000 | 3459.0 | 18.68% | 2307.8 | 7.32% | motif file (matrix) | svg |
| 5 | C T A G T C G A G C A T C A T G G C T A T A G C C G A T G T A C C T G A A G C T | JunB(bZIP)/DendriticCells-Junb-ChIP-Seq(GSE36099)/Homer | 1e-538 | -1.240e+03 | 0.0000 | 3163.0 | 17.08% | 2027.2 | 6.43% | motif file (matrix) | svg |
| 6 | C A G T T G C A A C G T A C T G C G T A A T C G C G A T T G A C C G T A A C G T | BATF(bZIP)/Th17-BATF-ChIP-Seq(GSE39756)/Homer | 1e-535 | -1.233e+03 | 0.0000 | 3557.0 | 19.21% | 2461.6 | 7.81% | motif file (matrix) | svg |
| 7 | T C G A A C G T C A T G G C T A T A G C C G A T G T A C G C T A A C G T A T G C | AP-1(bZIP)/ThioMac-PU.1-ChIP-Seq(GSE21512)/Homer | 1e-509 | -1.173e+03 | 0.0000 | 3941.0 | 21.28% | 2967.7 | 9.41% | motif file (matrix) | svg |
| 8 | C T A G T C G A C G A T A C T G C G T A T A C G A G C T T G A C G C T A A C G T G A T C T A G C | Fosl2(bZIP)/3T3L1-Fosl2-ChIP-Seq(GSE56872)/Homer | 1e-509 | -1.173e+03 | 0.0000 | 2117.0 | 11.43% | 1055.6 | 3.35% | motif file (matrix) | svg |
| 9 | C T A G T C G A A C G T A C T G C G T A T A G C C G A T G T A C C G T A A G C T G A T C G T A C | Jun-AP1(bZIP)/K562-cJun-ChIP-Seq(GSE31477)/Homer | 1e-486 | -1.120e+03 | 0.0000 | 1656.0 | 8.94% | 695.6 | 2.21% | motif file (matrix) | svg |
| 10 | C G A T T A C G T G A C G A C T C A T G C G T A T A C G A C G T G T A C C T G A | Bach2(bZIP)/OCILy7-Bach2-ChIP-Seq(GSE44420)/Homer | 1e-273 | -6.293e+02 | 0.0000 | 1271.0 | 6.86% | 677.6 | 2.15% | motif file (matrix) | svg |
| 11 | G T C A G C A T A C T G G T A C G A C T A C T G G C T A A T C G C A G T G T A C C G T A A G C T | Nrf2(bZIP)/Lymphoblast-Nrf2-ChIP-Seq(GSE37589)/Homer | 1e-172 | -3.980e+02 | 0.0000 | 473.0 | 2.55% | 158.6 | 0.50% | motif file (matrix) | svg |
| 12 | C G T A C G T A C G T A G C A T G C A T T A C G G T A C G A C T A C T G C G T A A T C G A C G T G T A C C G T A A G C T | Bach1(bZIP)/K562-Bach1-ChIP-Seq(GSE31477)/Homer | 1e-157 | -3.621e+02 | 0.0000 | 541.0 | 2.92% | 225.5 | 0.72% | motif file (matrix) | svg |
| 13 | T A C G T C G A C A G T A C T G G C T A A T G C C G A T G T A C C G T A A C T G T A G C C G T A | NF-E2(bZIP)/K562-NFE2-ChIP-Seq(GSE31477)/Homer | 1e-153 | -3.537e+02 | 0.0000 | 512.0 | 2.77% | 207.4 | 0.66% | motif file (matrix) | svg |
| 14 | C G T A C G T A C G T A G C A T G C A T A C T G G T A C G A C T C T A G G C T A T A C G G A C T T G A C C G T A A G C T | NFE2L2(bZIP)/HepG2-NFE2L2-ChIP-Seq(Encode)/Homer | 1e-106 | -2.457e+02 | 0.0000 | 458.0 | 2.47% | 228.8 | 0.73% | motif file (matrix) | svg |
| 15 | A T G C A G T C C T G A A G T C C G A T A C G T A G T C A G T C A C G T A T C G G A C T A C G T | Etv2(ETS)/ES-ER71-ChIP-Seq(GSE59402)/Homer | 1e-106 | -2.444e+02 | 0.0000 | 3080.0 | 16.63% | 3542.0 | 11.23% | motif file (matrix) | svg |
| 16 | A G T C C T G A A G T C C G A T C A G T G A T C A T G C A C T G A T C G G A C T | Fli1(ETS)/CD8-FLI-ChIP-Seq(GSE20898)/Homer | 1e-92 | -2.129e+02 | 0.0000 | 3285.0 | 17.74% | 3944.0 | 12.51% | motif file (matrix) | svg |
| 17 | T C G A T A G C G T C A A C T G A C T G C G T A C G T A C T A G A G C T T C A G | ERG(ETS)/VCaP-ERG-ChIP-Seq(GSE14097)/Homer | 1e-90 | -2.089e+02 | 0.0000 | 4997.0 | 26.99% | 6543.4 | 20.75% | motif file (matrix) | svg |
| 18 | T C A G T A G C G A C T C A T G C T G A A T C G G C A T G T A C C G T A A C T G T A G C T G C A | MafK(bZIP)/C2C12-MafK-ChIP-Seq(GSE36030)/Homer | 1e-90 | -2.084e+02 | 0.0000 | 962.0 | 5.20% | 799.9 | 2.54% | motif file (matrix) | svg |
| 19 | T C G A T A G C T G C A A C T G A C T G C G T A C G T A C T A G G A C T T A C G | ETS1(ETS)/Jurkat-ETS1-ChIP-Seq(GSE17954)/Homer | 1e-83 | -1.913e+02 | 0.0000 | 3316.0 | 17.91% | 4068.3 | 12.90% | motif file (matrix) | svg |
| 20 | T C G A C T G A T A G C T G A C T C A G T C A G C G T A C G T A T C A G A G C T | ETV1(ETS)/GIST48-ETV1-ChIP-Seq(GSE22441)/Homer | 1e-78 | -1.811e+02 | 0.0000 | 3942.0 | 21.29% | 5048.7 | 16.01% | motif file (matrix) | svg |
| 21 | C T G A T A G C T G A C T C A G C T A G G T C A C G T A T C A G A G C T T C A G | ETV4(ETS)/HepG2-ETV4-ChIP-Seq(ENCODE)/Homer | 1e-75 | -1.735e+02 | 0.0000 | 3022.0 | 16.32% | 3702.2 | 11.74% | motif file (matrix) | svg |
| 22 | T G C A C T G A A G T C G T C A A C T G A C T G C G T A C G T A C T G A A G C T | EWS:FLI1-fusion(ETS)/SK\_N\_MC-EWS:FLI1-ChIP-Seq(SRA014231)/Homer | 1e-71 | -1.640e+02 | 0.0000 | 1858.0 | 10.03% | 2063.5 | 6.54% | motif file (matrix) | svg |
| 23 | T C G A A G C T A C G T A C G T A G T C A G T C A C G T A T C G G A C T A T C G | EWS:ERG-fusion(ETS)/CADO\_ES1-EWS:ERG-ChIP-Seq(SRA014231)/Homer | 1e-70 | -1.620e+02 | 0.0000 | 2692.0 | 14.54% | 3261.3 | 10.34% | motif file (matrix) | svg |
| 24 | T C G A T C G A T A G C G T A C T C A G T A C G C G T A C G T A T C A G A G C T | GABPA(ETS)/Jurkat-GABPa-ChIP-Seq(GSE17954)/Homer | 1e-69 | -1.608e+02 | 0.0000 | 2549.0 | 13.77% | 3057.5 | 9.70% | motif file (matrix) | svg |
| 25 | G A T C T C G A A G T C C G A T C G A T A G T C A T G C A C T G A T C G G A C T | Elk1(ETS)/Hela-Elk1-ChIP-Seq(GSE31477)/Homer | 1e-55 | -1.272e+02 | 0.0000 | 1289.0 | 6.96% | 1387.9 | 4.40% | motif file (matrix) | svg |
| 26 | C T G A A G T C C G A T A G C T A T G C G T A C A C G T A T C G C A G T G C A T | Elf4(ETS)/BMDM-Elf4-ChIP-Seq(GSE88699)/Homer | 1e-51 | -1.190e+02 | 0.0000 | 2818.0 | 15.22% | 3627.6 | 11.50% | motif file (matrix) | svg |
| 27 | G A T C C T G A A G T C C G A T C G A T G A T C A G T C A C T G A T C G A G C T | Elk4(ETS)/Hela-Elk4-ChIP-Seq(GSE31477)/Homer | 1e-51 | -1.181e+02 | 0.0000 | 1305.0 | 7.05% | 1435.5 | 4.55% | motif file (matrix) | svg |
| 28 | T G C A C T G A A T G C G T C A A C T G A C T G C G T A C G T A C T A G A G C T | Ets1-distal(ETS)/CD4+-PolII-ChIP-Seq(Barski\_et\_al.)/Homer | 1e-48 | -1.117e+02 | 0.0000 | 1022.0 | 5.52% | 1069.7 | 3.39% | motif file (matrix) | svg |
| 29 | C G T A T A G C T A G C T G C A A C T G C T A G C G T A C G T A T C A G G A C T | EHF(ETS)/LoVo-EHF-ChIP-Seq(GSE49402)/Homer | 1e-47 | -1.103e+02 | 0.0000 | 4058.0 | 21.92% | 5581.2 | 17.70% | motif file (matrix) | svg |
| 30 | T G C A T C G A T A G C G T A C T C A G C T A G G T C A G C T A T C A G G A C T | ETS(ETS)/Promoter/Homer | 1e-44 | -1.020e+02 | 0.0000 | 734.0 | 3.96% | 715.4 | 2.27% | motif file (matrix) | svg |
| 31 | C G T A T G A C T A G C T G C A A C T G A C T G C G T A C G T A T C A G G A C T | ELF3(ETS)/PDAC-ELF3-ChIP-Seq(GSE64557)/Homer | 1e-43 | -9.964e+01 | 0.0000 | 2355.0 | 12.72% | 3022.7 | 9.59% | motif file (matrix) | svg |
| 32 | C T G A T G C A T A G C T G A C T A C G T C A G C T G A G C T A T C A G G A C T | ELF1(ETS)/Jurkat-ELF1-ChIP-Seq(SRA014231)/Homer | 1e-35 | -8.072e+01 | 0.0000 | 1075.0 | 5.81% | 1233.9 | 3.91% | motif file (matrix) | svg |
| 33 | G C T A A G T C T A C G T G C A A T C G T C A G G C T A T C G A T C A G A G C T | ELF5(ETS)/T47D-ELF5-ChIP-Seq(GSE30407)/Homer | 1e-31 | -7.165e+01 | 0.0000 | 2222.0 | 12.00% | 2963.7 | 9.40% | motif file (matrix) | svg |
| 34 | C G T A G A C T C G A T A T C G G T A C G C A T C A T G C G T A T A C G G C A T G T A C C G T A C A T G A T G C G C T A C T A G G C A T G C A T G C A T G A C T | MafB(bZIP)/BMM-Mafb-ChIP-Seq(GSE75722)/Homer | 1e-29 | -6.888e+01 | 0.0000 | 1677.0 | 9.06% | 2155.5 | 6.84% | motif file (matrix) | svg |
| 35 | T G A C A G T C C T G A T G A C C G T A A C G T A C G T A G T C A G T C C G T A | TEAD1(TEAD)/HepG2-TEAD1-ChIP-Seq(Encode)/Homer | 1e-27 | -6.279e+01 | 0.0000 | 2948.0 | 15.92% | 4142.8 | 13.14% | motif file (matrix) | svg |
| 36 | C T G A A T G C C G T A A C G T A G T C A G T C A C G T A C T G A T C G G C A T | SPDEF(ETS)/VCaP-SPDEF-ChIP-Seq(SRA014231)/Homer | 1e-26 | -6.043e+01 | 0.0000 | 3076.0 | 16.61% | 4360.2 | 13.83% | motif file (matrix) | svg |
| 37 | A G C T C T A G T G A C C G T A A C G T C G A T A G T C A G T C C T G A C A T G | TEAD3(TEA)/HepG2-TEAD3-ChIP-Seq(Encode)/Homer | 1e-23 | -5.472e+01 | 0.0000 | 3447.0 | 18.62% | 4993.0 | 15.83% | motif file (matrix) | svg |
| 38 | G A T C G T A C C G A T A C T G A C T G C G T A C G T A A C G T A C T G G A T C | TEAD(TEA)/Fibroblast-PU.1-ChIP-Seq(Unpublished)/Homer | 1e-23 | -5.459e+01 | 0.0000 | 2200.0 | 11.88% | 3031.7 | 9.61% | motif file (matrix) | svg |
| 39 | T A C G T G C A A G T C C G T A A C G T T G A C A C G T A C T G A C T G G C A T | TCF4(bHLH)/SHSY5Y-TCF4-ChIP-Seq(GSE96915)/Homer | 1e-23 | -5.451e+01 | 0.0000 | 5250.0 | 28.35% | 7909.5 | 25.08% | motif file (matrix) | svg |
| 40 | C T A G T C G A T G A C A G T C C G T A A C T G G T A C A C G T A C T G A C T G | BHLHA15(bHLH)/NIH3T3-BHLHB8.HA-ChIP-Seq(GSE119782)/Homer | 1e-22 | -5.291e+01 | 0.0000 | 4997.0 | 26.99% | 7511.1 | 23.82% | motif file (matrix) | svg |
| 41 | G A C T C G A T C T G A G T C A G A C T C G A T T C G A C G T A G C T A G C T A T G A C G T A C C G T A A C T G T G C A C G A T A C T G A C G T | Pitx1:Ebox(Homeobox,bHLH)/Hindlimb-Pitx1-ChIP-Seq(GSE41591)/Homer | 1e-19 | -4.492e+01 | 0.0000 | 911.0 | 4.92% | 1135.3 | 3.60% | motif file (matrix) | svg |
| 42 | T A G C T A G C G C A T C A T G A C T G G C T A C G T A A C G T A C T G G A T C | TEAD4(TEA)/Tropoblast-Tead4-ChIP-Seq(GSE37350)/Homer | 1e-19 | -4.483e+01 | 0.0000 | 2471.0 | 13.35% | 3523.3 | 11.17% | motif file (matrix) | svg |
| 43 | C G T A T A C G T C G A A C T G A C T G C G T A C G T A T A C G A G C T T A C G | PU.1(ETS)/ThioMac-PU.1-ChIP-Seq(GSE21512)/Homer | 1e-19 | -4.483e+01 | 0.0000 | 1415.0 | 7.64% | 1887.9 | 5.99% | motif file (matrix) | svg |
| 44 | T G A C A G T C C G T A A C T G G T A C A C G T A C T G A C G T G A C T G A T C | Twist2(bHLH)/Myoblast-Twist2.Ty1-ChIP-Seq(GSE127998)/Homer | 1e-18 | -4.284e+01 | 0.0000 | 6159.0 | 33.26% | 9531.3 | 30.23% | motif file (matrix) | svg |
| 45 | T C G A T G A C G T A C C G T A C A G T T G A C A C G T A C T G A G C T A G C T | NeuroG2(bHLH)/Fibroblast-NeuroG2-ChIP-Seq(GSE75910)/Homer | 1e-16 | -3.871e+01 | 0.0000 | 5337.0 | 28.82% | 8220.8 | 26.07% | motif file (matrix) | svg |
| 46 | A T G C G A T C C G A T C T A G A C T G G C T A C G T A A G C T A C T G A G C T | TEAD2(TEA)/Py2T-Tead2-ChIP-Seq(GSE55709)/Homer | 1e-16 | -3.731e+01 | 0.0000 | 1511.0 | 8.16% | 2080.1 | 6.60% | motif file (matrix) | svg |
| 47 | G A C T C T A G G A T C C A G T A C T G C T G A A T G C G C A T A T G C C T G A | MafA(bZIP)/Islet-MafA-ChIP-Seq(GSE30298)/Homer | 1e-15 | -3.580e+01 | 0.0000 | 2956.0 | 15.96% | 4372.4 | 13.87% | motif file (matrix) | svg |
| 48 | T C G A G C A T A C T G C T G A A G T C T C A G G A C T G T A C C G T A A G C T A G T C G A T C | c-Jun-CRE(bZIP)/K562-cJun-ChIP-Seq(GSE31477)/Homer | 1e-14 | -3.288e+01 | 0.0000 | 849.0 | 4.59% | 1101.2 | 3.49% | motif file (matrix) | svg |
| 49 | T C A G A C G T T C G A T A G C A G T C C G T A A C T G G T A C A C G T A C T G A T C G A G T C | Atoh1(bHLH)/Cerebellum-Atoh1-ChIP-Seq(GSE22111)/Homer | 1e-13 | -3.075e+01 | 0.0000 | 3770.0 | 20.36% | 5743.2 | 18.21% | motif file (matrix) | svg |
| 50 | C T G A T C A G G T A C G C T A A C T G T G A C G C A T C A T G | SCL(bHLH)/HPC7-Scl-ChIP-Seq(GSE13511)/Homer | 1e-13 | -3.032e+01 | 0.0000 | 13555.0 | 73.21% | 22310.8 | 70.75% | motif file (matrix) | svg |
| 51 | A G T C G A C T C A G T A C T G C T A G T G A C G C T A A T G C G C A T A T C G C G A T A C T G G A T C G T A C G T C A C T G A | NF1(CTF)/LNCAP-NF1-ChIP-Seq(Unpublished)/Homer | 1e-13 | -3.025e+01 | 0.0000 | 887.0 | 4.79% | 1172.3 | 3.72% | motif file (matrix) | svg |
| 52 | T A C G C T G A T C G A C G A T C T A G C T A G T C G A C T G A T C G A T C G A C G T A T C G A G C A T C A T G C G T A T A C G G C A T T G A C C G T A A G C T | NFAT:AP1(RHD,bZIP)/Jurkat-NFATC1-ChIP-Seq(Jolma\_et\_al.)/Homer | 1e-13 | -3.018e+01 | 0.0000 | 633.0 | 3.42% | 795.9 | 2.52% | motif file (matrix) | svg |
| 53 | T G C A T C G A T C G A A C G T C A T G C G T A A G T C T C A G A C G T G T A C C G T A A G C T | CREB5(bZIP)/LNCaP-CREB5.V5-ChIP-Seq(GSE137775)/Homer | 1e-12 | -2.960e+01 | 0.0000 | 1141.0 | 6.16% | 1562.5 | 4.96% | motif file (matrix) | svg |
| 54 | A T G C T C A G T C G A G C A T A C T G C G T A A G T C T C A G G A C T T G A C C G T A A G C T | Atf2(bZIP)/3T3L1-Atf2-ChIP-Seq(GSE56872)/Homer | 1e-12 | -2.898e+01 | 0.0000 | 861.0 | 4.65% | 1140.6 | 3.62% | motif file (matrix) | svg |
| 55 | T A G C C T A G T C G A G A C T A C T G C T G A A G T C T C A G G C A T T G A C C T G A A G C T | Atf7(bZIP)/3T3L1-Atf7-ChIP-Seq(GSE56872)/Homer | 1e-12 | -2.767e+01 | 0.0000 | 1226.0 | 6.62% | 1706.6 | 5.41% | motif file (matrix) | svg |
| 56 | G C T A C T G A T C G A A G T C A G T C C T G A A G T C G T C A C T G A T G C A | RUNX1(Runt)/Jurkat-RUNX1-ChIP-Seq(GSE29180)/Homer | 1e-11 | -2.695e+01 | 0.0000 | 2894.0 | 15.63% | 4363.8 | 13.84% | motif file (matrix) | svg |
| 57 | A G T C G A C T C A G T G T A C A G T C A T C G T C A G A C T G G T C A C G T A | Stat3(Stat)/mES-Stat3-ChIP-Seq(GSE11431)/Homer | 1e-11 | -2.618e+01 | 0.0000 | 1270.0 | 6.86% | 1785.0 | 5.66% | motif file (matrix) | svg |
| 58 | C G A T T G C A G T A C C G T A A G T C C T A G G A C T C A T G | NPAS(bHLH)/Liver-NPAS-ChIP-Seq(GSE39860)/Homer | 1e-11 | -2.608e+01 | 0.0000 | 4263.0 | 23.02% | 6609.7 | 20.96% | motif file (matrix) | svg |
| 59 | C G A T T G C A T G C A G A T C C G T A A C T G T G A C G A C T C A T G A C T G | Tcf21(bHLH)/ArterySmoothMuscle-Tcf21-ChIP-Seq(GSE61369)/Homer | 1e-10 | -2.498e+01 | 0.0000 | 3651.0 | 19.72% | 5619.4 | 17.82% | motif file (matrix) | svg |
| 60 | T C A G T G A C G T A C C G T A A C G T T G A C A C G T T C A G A G C T G A C T | NeuroD1(bHLH)/Islet-NeuroD1-ChIP-Seq(GSE30298)/Homer | 1e-9 | -2.287e+01 | 0.0000 | 2591.0 | 13.99% | 3919.3 | 12.43% | motif file (matrix) | svg |
| 61 | T C G A G C A T A C G T C T A G G T A C T C G A G C A T T G A C T C G A A C G T | Chop(bZIP)/MEF-Chop-ChIP-Seq(GSE35681)/Homer | 1e-9 | -2.209e+01 | 0.0000 | 937.0 | 5.06% | 1299.5 | 4.12% | motif file (matrix) | svg |
| 62 | C G A T C T G A C G T A C A G T A G T C G A T C G A T C A C T G | Pitx1(Homeobox)/Chicken-Pitx1-ChIP-Seq(GSE38910)/Homer | 1e-9 | -2.190e+01 | 0.0000 | 11302.0 | 61.04% | 18543.3 | 58.80% | motif file (matrix) | svg |
| 63 | T G C A C T G A A T G C G T C A A C G T A T G C A C G T A C T G A C T G T G C A | ZBTB18(Zf)/HEK293-ZBTB18.GFP-ChIP-Seq(GSE58341)/Homer | 1e-9 | -2.074e+01 | 0.0000 | 1891.0 | 10.21% | 2816.0 | 8.93% | motif file (matrix) | svg |
| 64 | C T G A T G A C T G A C C G T A A C G T T G A C A G C T C T A G A C G T G A C T | Olig2(bHLH)/Neuron-Olig2-ChIP-Seq(GSE30882)/Homer | 1e-8 | -2.071e+01 | 0.0000 | 6358.0 | 34.34% | 10174.6 | 32.27% | motif file (matrix) | svg |
| 65 | A T G C T C G A A G T C A G C T A C G T G T A C A G T C G C T A C T A G C A T G G T C A C T G A T C A G A G T C | Stat3+il21(Stat)/CD4-Stat3-ChIP-Seq(GSE19198)/Homer | 1e-8 | -2.024e+01 | 0.0000 | 2006.0 | 10.83% | 3005.9 | 9.53% | motif file (matrix) | svg |
| 66 | C T G A A C T G A C T G A G T C A G T C A G C T C T A G T A C G | ZFX(Zf)/mES-Zfx-ChIP-Seq(GSE11431)/Homer | 1e-8 | -1.989e+01 | 0.0000 | 2116.0 | 11.43% | 3186.8 | 10.11% | motif file (matrix) | svg |
| 67 | T C G A A C G T A C T G C T G A A G T C T C A G A G C T G T A C C G T A A G C T G A T C T C G A | JunD(bZIP)/K562-JunD-ChIP-Seq/Homer | 1e-8 | -1.968e+01 | 0.0000 | 238.0 | 1.29% | 271.9 | 0.86% | motif file (matrix) | svg |
| 68 | C T A G T C A G C A G T T C A G A C T G A C T G G A T C C T A G A C T G C T A G T C A G A T G C | KLF14(Zf)/HEK293-KLF14.GFP-ChIP-Seq(GSE58341)/Homer | 1e-8 | -1.919e+01 | 0.0000 | 2814.0 | 15.20% | 4327.7 | 13.72% | motif file (matrix) | svg |
| 69 | T G C A A G C T C A T G C G T A A G C T A C T G G A T C G T C A C G T A A G C T | Atf4(bZIP)/MEF-Atf4-ChIP-Seq(GSE35681)/Homer | 1e-8 | -1.916e+01 | 0.0000 | 1202.0 | 6.49% | 1735.5 | 5.50% | motif file (matrix) | svg |
| 70 | T A G C A G T C T G A C A G T C C T A G A T C G A G T C C A T G T G A C A G T C G T A C A G T C A G T C G C A T C T A G A T C G G C A T A C T G A T C G G A T C | BORIS(Zf)/K562-CTCFL-ChIP-Seq(GSE32465)/Homer | 1e-8 | -1.848e+01 | 0.0000 | 452.0 | 2.44% | 585.5 | 1.86% | motif file (matrix) | svg |
| 71 | T C G A A C T G A C T G C G T A C G T A T C G A A G T C C T G A A T C G G T A C G C A T C A T G | ETS:E-box(ETS,bHLH)/HPC7-Scl-ChIP-Seq(GSE22178)/Homer | 1e-7 | -1.791e+01 | 0.0000 | 281.0 | 1.52% | 338.3 | 1.07% | motif file (matrix) | svg |
| 72 | T A C G T A C G G T A C A T C G T A C G T A C G G T C A C T G A C G T A G A C T | E2F4(E2F)/K562-E2F4-ChIP-Seq(GSE31477)/Homer | 1e-7 | -1.778e+01 | 0.0000 | 645.0 | 3.48% | 880.2 | 2.79% | motif file (matrix) | svg |
| 73 | T G C A A G C T C T G A A T C G G A C T C T A G G T A C G A T C G T C A A G T C G T A C G A C T C T A G A T C G G C A T C A T G C A T G G A T C G T A C C T G A | CTCF(Zf)/CD4+-CTCF-ChIP-Seq(Barski\_et\_al.)/Homer | 1e-7 | -1.773e+01 | 0.0000 | 291.0 | 1.57% | 353.5 | 1.12% | motif file (matrix) | svg |
| 74 | T A G C G C T A T C G A C T G A A G T C A G T C C T G A A G T C C G T A C T A G | RUNX(Runt)/HPC7-Runx1-ChIP-Seq(GSE22178)/Homer | 1e-7 | -1.765e+01 | 0.0000 | 1891.0 | 10.21% | 2849.8 | 9.04% | motif file (matrix) | svg |
| 75 | C G A T C T A G A C G T A C G T A C G T C G T A A G C T C G A T A G C T C G T A C T A G T A G C | FoxD3(forkhead)/ZebrafishEmbryo-Foxd3.biotin-ChIP-seq(GSE106676)/Homer | 1e-7 | -1.722e+01 | 0.0000 | 3007.0 | 16.24% | 4670.6 | 14.81% | motif file (matrix) | svg |
| 76 | C T A G C A G T T G A C C G T A G A T C T C A G G A C T C A T G | BMAL1(bHLH)/Liver-Bmal1-ChIP-Seq(GSE39860)/Homer | 1e-7 | -1.707e+01 | 0.0000 | 4566.0 | 24.66% | 7247.9 | 22.98% | motif file (matrix) | svg |
| 77 | T C G A T G A C A G T C C G T A A G T C C T A G A C G T A C T G A C T G A G C T A G T C G C A T | Max(bHLH)/K562-Max-ChIP-Seq(GSE31477)/Homer | 1e-7 | -1.705e+01 | 0.0000 | 1608.0 | 8.68% | 2401.8 | 7.62% | motif file (matrix) | svg |
| 78 | T C G A T A G C G T C A A C T G C T A G C G T A C G A T A C T G A C G T A C T G A C T G A C G T | ETS:RUNX(ETS,Runt)/Jurkat-RUNX1-ChIP-Seq(GSE17954)/Homer | 1e-7 | -1.697e+01 | 0.0000 | 222.0 | 1.20% | 258.7 | 0.82% | motif file (matrix) | svg |
| 79 | A C T G G A T C G A C T A C T G A C G T C A T G A C T G A C G T A G C T C G A T | RUNX-AML(Runt)/CD4+-PolII-ChIP-Seq(Barski\_et\_al.)/Homer | 1e-7 | -1.695e+01 | 0.0000 | 2153.0 | 11.63% | 3281.7 | 10.41% | motif file (matrix) | svg |
| 80 | A T G C C T G A G A C T A C G T A C G T G T A C G A T C C G A T C T A G C A T G C G T A C G T A C T G A G A C T | STAT1(Stat)/HelaS3-STAT1-ChIP-Seq(GSE12782)/Homer | 1e-7 | -1.684e+01 | 0.0000 | 859.0 | 4.64% | 1217.7 | 3.86% | motif file (matrix) | svg |
| 81 | G A C T C G A T T C A G G A T C G A C T A G C T A G C T A G T C G A T C C G T A C T A G C T A G T C G A T C G A C T G A | Bcl6(Zf)/Liver-Bcl6-ChIP-Seq(GSE31578)/Homer | 1e-7 | -1.637e+01 | 0.0000 | 3720.0 | 20.09% | 5857.3 | 18.57% | motif file (matrix) | svg |
| 82 | T A G C C G A T A C G T A G C T A G C T A G T C A T G C A G T C A C T G A T G C A T G C G C T A | E2F7(E2F)/Hela-E2F7-ChIP-Seq(GSE32673)/Homer | 1e-6 | -1.594e+01 | 0.0000 | 141.0 | 0.76% | 150.0 | 0.48% | motif file (matrix) | svg |
| 83 | G T C A T G C A G C T A A G T C C G T A A C T G T G A C G C A T T C A G C A G T | Ap4(bHLH)/AML-Tfap4-ChIP-Seq(GSE45738)/Homer | 1e-6 | -1.586e+01 | 0.0000 | 4636.0 | 25.04% | 7385.0 | 23.42% | motif file (matrix) | svg |
| 84 | T A C G T C G A T A G C A G T C C G T A A G T C C T A G G C A T A C T G A T C G | n-Myc(bHLH)/mES-nMyc-ChIP-Seq(GSE11431)/Homer | 1e-6 | -1.549e+01 | 0.0000 | 1727.0 | 9.33% | 2610.3 | 8.28% | motif file (matrix) | svg |
| 85 | T C G A A C G T A C G T C T G A G A T C T C A G G A C T G T C A C G T A A G C T G T C A C T A G A G C T A C G T T C G A | NFIL3(bZIP)/HepG2-NFIL3-ChIP-Seq(Encode)/Homer | 1e-6 | -1.533e+01 | 0.0000 | 2025.0 | 10.94% | 3094.1 | 9.81% | motif file (matrix) | svg |
| 86 | T A C G T C G A G A C T A C T G C T G A A G T C T C A G G A C T T G A C C T G A | Atf1(bZIP)/K562-ATF1-ChIP-Seq(GSE31477)/Homer | 1e-6 | -1.533e+01 | 0.0000 | 1763.0 | 9.52% | 2670.8 | 8.47% | motif file (matrix) | svg |
| 87 | T C A G A G C T A C G T A C G T G T A C G A T C C G T A C T A G C A T G G T C A C G T A T C G A | STAT4(Stat)/CD4-Stat4-ChIP-Seq(GSE22104)/Homer | 1e-6 | -1.497e+01 | 0.0000 | 2971.0 | 16.05% | 4645.4 | 14.73% | motif file (matrix) | svg |
| 88 | G A C T G T A C T G C A A C G T G A T C G C T A T C G A A C G T A G T C C G T A | Pdx1(Homeobox)/Islet-Pdx1-ChIP-Seq(SRA008281)/Homer | 1e-6 | -1.484e+01 | 0.0000 | 3217.0 | 17.37% | 5052.5 | 16.02% | motif file (matrix) | svg |
| 89 | A C T G C A T G G C T A T C G A G C T A A G C T A G C T G T A C A G T C T G A C | NFkB-p65-Rel(RHD)/ThioMac-LPS-Expression(GSE23622)/Homer | 1e-6 | -1.480e+01 | 0.0000 | 193.0 | 1.04% | 226.0 | 0.72% | motif file (matrix) | svg |
| 90 | T G A C C T A G T C A G G T C A C G T A T C A G C G A T T C A G T C G A T G C A C T G A T A G C | PU.1-IRF(ETS:IRF)/Bcell-PU.1-ChIP-Seq(GSE21512)/Homer | 1e-6 | -1.465e+01 | 0.0000 | 4062.0 | 21.94% | 6453.0 | 20.46% | motif file (matrix) | svg |
| 91 | T G A C G C T A T C G A T G C A A G T C A G T C C G T A A G T C C G T A C T G A G C T A G T A C | RUNX2(Runt)/PCa-RUNX2-ChIP-Seq(GSE33889)/Homer | 1e-6 | -1.456e+01 | 0.0000 | 2319.0 | 12.52% | 3583.8 | 11.36% | motif file (matrix) | svg |
| 92 | G T A C C A G T A C T G A C T G A C T G G A T C A C T G A C G T A C T G A C T G A G T C G A T C | KLF6(Zf)/PDAC-KLF6-ChIP-Seq(GSE64557)/Homer | 1e-6 | -1.445e+01 | 0.0000 | 1674.0 | 9.04% | 2537.8 | 8.05% | motif file (matrix) | svg |
| 93 | C T A G T C G A C G A T C T A G G C A T C A G T C T A G G A T C C G T A G T C A | CEBP:AP1(bZIP)/ThioMac-CEBPb-ChIP-Seq(GSE21512)/Homer | 1e-6 | -1.444e+01 | 0.0000 | 2868.0 | 15.49% | 4484.3 | 14.22% | motif file (matrix) | svg |
| 94 | C G T A C A T G C A T G A C T G C T A G T C G A G C A T C G A T A G C T A G T C G A T C G T A C | NFkB-p65(RHD)/GM12787-p65-ChIP-Seq(GSE19485)/Homer | 1e-6 | -1.425e+01 | 0.0000 | 1353.0 | 7.31% | 2023.6 | 6.42% | motif file (matrix) | svg |
| 95 | C G T A C T G A C T A G C G T A C G T A A G T C C G T A C A G T G C A T G T C A C G A T A C T G A C G T G C A T G A T C | PGR(NR)/EndoStromal-PGR-ChIP-Seq(GSE69539)/Homer | 1e-6 | -1.396e+01 | 0.0000 | 810.0 | 4.37% | 1164.4 | 3.69% | motif file (matrix) | svg |
| 96 | T G A C G T A C C G T A A C T G T G A C C G A T A C T G A T C G A G C T T A C G T C G A T A G C G T A C C G T A A T C G T G A C G C A T A C T G A C T G A T G C | Twist(bHLH)/HMLE-TWIST1-ChIP-Seq(Chang\_et\_al)/Homer | 1e-5 | -1.349e+01 | 0.0000 | 497.0 | 2.68% | 682.7 | 2.16% | motif file (matrix) | svg |
| 97 | T A G C C G A T T A C G A C T G A G T C A C T G A T C G A T C G C G T A C T G A | E2F1(E2F)/Hela-E2F1-ChIP-Seq(GSE22478)/Homer | 1e-5 | -1.313e+01 | 0.0000 | 333.0 | 1.80% | 436.6 | 1.38% | motif file (matrix) | svg |
| 98 | T G C A A C T G T A C G A T G C A G T C G A C T T C G A A T C G | ZNF711(Zf)/SHSY5Y-ZNF711-ChIP-Seq(GSE20673)/Homer | 1e-5 | -1.304e+01 | 0.0000 | 2890.0 | 15.61% | 4543.9 | 14.41% | motif file (matrix) | svg |
| 99 | T A C G T C A G A G C T A T G C C G T A A G T C T C A G A C G T A C T G T C G A | USF1(bHLH)/GM12878-Usf1-ChIP-Seq(GSE32465)/Homer | 1e-5 | -1.299e+01 | 0.0000 | 1034.0 | 5.58% | 1527.6 | 4.84% | motif file (matrix) | svg |
| 100 | C T A G C G T A G T C A C G T A A G T C G A T C A G C T C T A G C G T A A C G T G T C A G A T C | Six2(Homeobox)/NephronProgenitor-Six2-ChIP-Seq(GSE39837)/Homer | 1e-5 | -1.299e+01 | 0.0000 | 2664.0 | 14.39% | 4173.0 | 13.23% | motif file (matrix) | svg |
| 101 | C A T G A G T C A G C T C G T A C G A T C G A T G C A T G C A T C G A T C T G A C A T G T G A C | Mef2d(MADS)/Retina-Mef2d-ChIP-Seq(GSE61391)/Homer | 1e-5 | -1.289e+01 | 0.0000 | 629.0 | 3.40% | 890.1 | 2.82% | motif file (matrix) | svg |
| 102 | A T G C G A C T A C T G C A G T G A T C A C G T T A C G T A C G | Smad2(MAD)/ES-SMAD2-ChIP-Seq(GSE29422)/Homer | 1e-5 | -1.266e+01 | 0.0000 | 4040.0 | 21.82% | 6454.9 | 20.47% | motif file (matrix) | svg |
| 103 | T G A C C G A T A C T G A C T G A C T G G A C T A C T G A C G T A C T G A C T G G A T C G A T C | EKLF(Zf)/Erythrocyte-Klf1-ChIP-Seq(GSE20478)/Homer | 1e-5 | -1.219e+01 | 0.0000 | 218.0 | 1.18% | 271.3 | 0.86% | motif file (matrix) | svg |
| 104 | T C A G G A C T C A G T C T G A A G C T C T A G G A C T T G C A C T G A A G T C | HLF(bZIP)/HSC-HLF.Flag-ChIP-Seq(GSE69817)/Homer | 1e-5 | -1.162e+01 | 0.0000 | 2769.0 | 14.95% | 4368.5 | 13.85% | motif file (matrix) | svg |
| 105 | G A C T C A G T A G C T C G A T A G T C G A T C A G T C C G T A A T G C T C A G | Rbpj1(?)/Panc1-Rbpj1-ChIP-Seq(GSE47459)/Homer | 1e-5 | -1.158e+01 | 0.0000 | 3849.0 | 20.79% | 6158.4 | 19.53% | motif file (matrix) | svg |
| 106 | G C T A T C G A C G T A C T A G A G C T G T C A G T C A C G T A A G T C C G T A | FOXA1(Forkhead)/LNCAP-FOXA1-ChIP-Seq(GSE27824)/Homer | 1e-5 | -1.154e+01 | 0.0000 | 3982.0 | 21.51% | 6380.1 | 20.23% | motif file (matrix) | svg |
| 107 | A G T C A C G T A C T G A G C T A C G T A C G T G T C A A G T C | Foxo1(Forkhead)/RAW-Foxo1-ChIP-Seq(Fan\_et\_al.)/Homer | 1e-4 | -1.132e+01 | 0.0001 | 5625.0 | 30.38% | 9133.8 | 28.96% | motif file (matrix) | svg |
| 108 | T C A G A G C T A T G C C G T A A G C T T C A G C A G T A C T G C T G A A G T C | MITF(bHLH)/MastCells-MITF-ChIP-Seq(GSE48085)/Homer | 1e-4 | -1.127e+01 | 0.0001 | 2062.0 | 11.14% | 3212.8 | 10.19% | motif file (matrix) | svg |
| 109 | C T A G A C T G C T A G T C A G T C A G T A C G C T A G A C T G | Maz(Zf)/HepG2-Maz-ChIP-Seq(GSE31477)/Homer | 1e-4 | -1.078e+01 | 0.0001 | 2323.0 | 12.55% | 3649.0 | 11.57% | motif file (matrix) | svg |
| 110 | T A C G A T C G T A G C G A T C A C T G A C G T A G T C A C G T C T A G A T C G | Smad4(MAD)/ESC-SMAD4-ChIP-Seq(GSE29422)/Homer | 1e-4 | -1.078e+01 | 0.0001 | 3979.0 | 21.49% | 6391.2 | 20.27% | motif file (matrix) | svg |
| 111 | A G T C G C A T C G T A C G T A G T A C A C G T A C T G G A T C G A T C T C G A | BMYB(HTH)/Hela-BMYB-ChIP-Seq(GSE27030)/Homer | 1e-4 | -1.049e+01 | 0.0001 | 4889.0 | 26.40% | 7918.2 | 25.11% | motif file (matrix) | svg |
| 112 | C T G A T C A G G T A C T G C A A G T C C G T A A G T C A C T G A C G T A C T G | MNT(bHLH)/HepG2-MNT-ChIP-Seq(Encode)/Homer | 1e-4 | -1.034e+01 | 0.0001 | 2512.0 | 13.57% | 3967.1 | 12.58% | motif file (matrix) | svg |
| 113 | C T G A T C A G C A G T C T A G A C T G C T A G G A T C A T C G A C T G C T G A T C A G G A T C | Sp5(Zf)/mES-Sp5.Flag-ChIP-Seq(GSE72989)/Homer | 1e-4 | -1.011e+01 | 0.0002 | 1471.0 | 7.94% | 2265.3 | 7.18% | motif file (matrix) | svg |
| 114 | T C A G C T A G C T A G T G C A C G A T C G A T C G T A C T A G | GSC(Homeobox)/FrogEmbryos-GSC-ChIP-Seq(DRA000576)/Homer | 1e-4 | -1.002e+01 | 0.0002 | 3272.0 | 17.67% | 5231.4 | 16.59% | motif file (matrix) | svg |
| 115 | C T A G C T A G A G T C T C A G A C T G A C G T A C G T C T G A | MYB(HTH)/ERMYB-Myb-ChIPSeq(GSE22095)/Homer | 1e-4 | -9.987e+00 | 0.0002 | 5476.0 | 29.57% | 8915.7 | 28.27% | motif file (matrix) | svg |
| 116 | A G C T G C A T A C T G A C G T A G T C A C G T C T A G T A C G | Smad3(MAD)/NPC-Smad3-ChIP-Seq(GSE36673)/Homer | 1e-4 | -9.802e+00 | 0.0002 | 7207.0 | 38.92% | 11838.9 | 37.54% | motif file (matrix) | svg |
| 117 | C A G T T C A G G A T C A C T G A C G T C T A G A C T G A C T G G A C T C T A G | Egr1(Zf)/K562-Egr1-ChIP-Seq(GSE32465)/Homer | 1e-4 | -9.622e+00 | 0.0002 | 1151.0 | 6.22% | 1754.0 | 5.56% | motif file (matrix) | svg |
| 118 | C G T A G C T A C G A T C T A G A C G T G T C A C G T A C G T A A G T C C G T A T G C A T A C G | FoxL2(Forkhead)/Ovary-FoxL2-ChIP-Seq(GSE60858)/Homer | 1e-4 | -9.531e+00 | 0.0003 | 2644.0 | 14.28% | 4200.6 | 13.32% | motif file (matrix) | svg |
| 119 | C G T A C T A G A C T G A C T G G A C T C T A G C A G T C T A G C A T G G A T C | KLF5(Zf)/LoVo-KLF5-ChIP-Seq(GSE49402)/Homer | 1e-3 | -9.194e+00 | 0.0004 | 1723.0 | 9.31% | 2690.1 | 8.53% | motif file (matrix) | svg |
| 120 | G T A C C G T A C G T A T A C G G C A T G T A C C G T A C A T G A G T C C G T A C G T A C G A T G C A T G C A T G A C T | MafF(bZIP)/HepG2-MafF-ChIP-Seq(GSE31477)/Homer | 1e-3 | -9.169e+00 | 0.0004 | 1096.0 | 5.92% | 1670.1 | 5.30% | motif file (matrix) | svg |
| 121 | A C G T T A C G G A T C A C T G A C G T C T A G A C T G A C T G G A T C C T A G C A T G C T A G | Egr2(Zf)/Thymocytes-Egr2-ChIP-Seq(GSE34254)/Homer | 1e-3 | -9.118e+00 | 0.0004 | 266.0 | 1.44% | 358.4 | 1.14% | motif file (matrix) | svg |
| 122 | G T A C G A T C C A G T A G T C A G T C A G T C T G C A G A T C C T G A A T G C G T C A A C G T | WT1(Zf)/Kidney-WT1-ChIP-Seq(GSE90016)/Homer | 1e-3 | -8.831e+00 | 0.0005 | 1197.0 | 6.46% | 1838.3 | 5.83% | motif file (matrix) | svg |
| 123 | G A C T C T A G C T A G A G T C T G C A A C T G A C G T A C G T C T A G T C A G | AMYB(HTH)/Testes-AMYB-ChIP-Seq(GSE44588)/Homer | 1e-3 | -8.641e+00 | 0.0006 | 4752.0 | 25.66% | 7734.3 | 24.53% | motif file (matrix) | svg |
| 124 | C A T G G T A C G A C T G C T A C G T A C G T A C G T A G C T A G A C T C T G A T C A G G T A C | Mef2c(MADS)/GM12878-Mef2c-ChIP-Seq(GSE32465)/Homer | 1e-3 | -8.541e+00 | 0.0007 | 1412.0 | 7.63% | 2192.9 | 6.95% | motif file (matrix) | svg |
| 125 | G C T A T A G C A G C T A T C G G T C A C G T A G C T A A T G C G A T C C T G A | IRF4(IRF)/GM12878-IRF4-ChIP-Seq(GSE32465)/Homer | 1e-3 | -8.537e+00 | 0.0007 | 1472.0 | 7.95% | 2290.8 | 7.26% | motif file (matrix) | svg |
| 126 | G T A C G A C T C G T A C T G A T C G A C G T A G C T A C A G T C T G A T A C G | Mef2a(MADS)/HL1-Mef2a.biotin-ChIP-Seq(GSE21529)/Homer | 1e-3 | -8.440e+00 | 0.0008 | 1249.0 | 6.75% | 1928.9 | 6.12% | motif file (matrix) | svg |
| 127 | T A C G A T C G G A T C G T C A C T G A G C A T C G A T G C T A T C G A G C T A | Unknown(Homeobox)/Limb-p300-ChIP-Seq/Homer | 1e-3 | -8.348e+00 | 0.0008 | 2215.0 | 11.96% | 3514.1 | 11.14% | motif file (matrix) | svg |
| 128 | A T C G G A C T C G A T C G T A C G T A C A G T G A T C G A T C G A T C A G C T | Otx2(Homeobox)/EpiLC-Otx2-ChIP-Seq(GSE56098)/Homer | 1e-3 | -8.239e+00 | 0.0009 | 2138.0 | 11.55% | 3390.0 | 10.75% | motif file (matrix) | svg |
| 129 | A G T C A T C G C T A G A G C T G A C T C T A G A G T C A G T C G C T A C A G T T C A G T C A G G A T C C T G A T C G A G A T C | RFX(HTH)/K562-RFX3-ChIP-Seq(SRA012198)/Homer | 1e-3 | -8.234e+00 | 0.0009 | 170.0 | 0.92% | 219.2 | 0.70% | motif file (matrix) | svg |
| 130 | T G A C C T G A C T A G C T G A C G T A A G T C C T G A A C G T G C A T T A G C G C A T A T C G G A C T G A C T G A T C | GRE(NR),IR3/RAW264.7-GRE-ChIP-Seq(Unpublished)/Homer | 1e-3 | -8.167e+00 | 0.0010 | 736.0 | 3.97% | 1103.9 | 3.50% | motif file (matrix) | svg |
| 131 | T C A G G C T A T C A G C A G T T G A C G T C A A G T C A T C G T G C A G T A C C A G T G A T C | Npas4(bHLH)/Neuron-Npas4-ChIP-Seq(GSE127793)/Homer | 1e-3 | -8.111e+00 | 0.0010 | 2085.0 | 11.26% | 3304.8 | 10.48% | motif file (matrix) | svg |
| 132 | T C A G T C A G T A G C A G T C C T G A A G T C C T A G A C G T A C T G A T C G | c-Myc(bHLH)/mES-cMyc-ChIP-Seq(GSE11431)/Homer | 1e-3 | -7.566e+00 | 0.0017 | 1214.0 | 6.56% | 1884.4 | 5.98% | motif file (matrix) | svg |
| 133 | A C T G C G T A A C T G A T G C T G A C G A T C A T C G T G C A A C T G A G T C | ZNF519(Zf)/HEK293-ZNF519.GFP-ChIP-Seq(GSE58341)/Homer | 1e-3 | -7.490e+00 | 0.0018 | 434.0 | 2.34% | 630.3 | 2.00% | motif file (matrix) | svg |
| 134 | T G C A C G T A G T C A A G C T A G T C G C T A T A G C C G A T C T A G G A T C | Gfi1b(Zf)/HPC7-Gfi1b-ChIP-Seq(GSE22178)/Homer | 1e-3 | -7.262e+00 | 0.0023 | 2360.0 | 12.75% | 3776.4 | 11.98% | motif file (matrix) | svg |
| 135 | C G T A A C G T A C G T A C G T A C G T A G T C A G T C C T G A A G C T A G C T | NFAT(RHD)/Jurkat-NFATC1-ChIP-Seq(Jolma\_et\_al.)/Homer | 1e-3 | -7.194e+00 | 0.0024 | 2917.0 | 15.75% | 4703.8 | 14.92% | motif file (matrix) | svg |
| 136 | G T A C C T A G T C A G A G C T T A G C C G T A A T G C T A C G A G T C G T A C G T C A A G T C | Srebp2(bHLH)/HepG2-Srebp2-ChIP-Seq(GSE31477)/Homer | 1e-3 | -7.142e+00 | 0.0026 | 312.0 | 1.69% | 442.8 | 1.40% | motif file (matrix) | svg |
| 137 | A G C T A C G T A C T G A T G C A G T C C G T A C T G A T A C G | NF1-halfsite(CTF)/LNCaP-NF1-ChIP-Seq(Unpublished)/Homer | 1e-3 | -7.040e+00 | 0.0028 | 5268.0 | 28.45% | 8646.8 | 27.42% | motif file (matrix) | svg |
| 138 | A T C G A G C T C T G A C T A G A C T G A C G T G T A C G C T A A T G C A C G T C T A G C A T G T A C G C G A T A T G C C G T A | Reverb(NR),DR2/RAW-Reverba.biotin-ChIP-Seq(GSE45914)/Homer | 1e-2 | -6.809e+00 | 0.0035 | 262.0 | 1.41% | 367.1 | 1.16% | motif file (matrix) | svg |
| 139 | A G T C G A T C A G T C C G T A A T C G C A G T A G T C G T A C C T G A A C T G T C A G A G C T A G C T A G C T A G C T | PRDM15(Zf)/ESC-Prdm15-ChIP-Seq(GSE73694)/Homer | 1e-2 | -6.777e+00 | 0.0036 | 2530.0 | 13.66% | 4069.3 | 12.90% | motif file (matrix) | svg |
| 140 | G C A T G C A T C T G A A C G T C T G A A C G T C G T A C G T A C G T A A G T C G T C A G T C A | Foxf1(Forkhead)/Lung-Foxf1-ChIP-Seq(GSE77951)/Homer | 1e-2 | -6.606e+00 | 0.0042 | 2879.0 | 15.55% | 4654.6 | 14.76% | motif file (matrix) | svg |
| 141 | A T C G A G C T A C T G A G T C A C T G A G T C C G T A A C G T A C T G A G T C A C T G A G T C | NRF(NRF)/Promoter/Homer | 1e-2 | -6.595e+00 | 0.0043 | 250.0 | 1.35% | 350.8 | 1.11% | motif file (matrix) | svg |
| 142 | A T C G G A T C G C A T C G T A G T C A A C G T A T G C A G T C | CRX(Homeobox)/Retina-Crx-ChIP-Seq(GSE20012)/Homer | 1e-2 | -6.594e+00 | 0.0043 | 6229.0 | 33.64% | 10281.8 | 32.61% | motif file (matrix) | svg |
| 143 | C T A G T A G C A G T C G T C A C T G A A C G T C G T A C G T A C G T A G C T A | Hoxd13(Homeobox)/ChickenMSG-Hoxd13.Flag-ChIP-Seq(GSE86088)/Homer | 1e-2 | -6.501e+00 | 0.0046 | 5394.0 | 29.13% | 8875.1 | 28.14% | motif file (matrix) | svg |
| 144 | G A C T C T A G C T A G C T A G A C T G T C G A C T G A C T A G C T A G C T A G G T A C G T C A | ZNF467(Zf)/HEK293-ZNF467.GFP-ChIP-Seq(GSE58341)/Homer | 1e-2 | -6.420e+00 | 0.0050 | 1938.0 | 10.47% | 3096.0 | 9.82% | motif file (matrix) | svg |
| 145 | T A C G T A C G G T A C A T C G A C T G T A C G T C G A C T G A T C G A A T C G | E2F6(E2F)/Hela-E2F6-ChIP-Seq(GSE31477)/Homer | 1e-2 | -6.332e+00 | 0.0054 | 922.0 | 4.98% | 1427.0 | 4.53% | motif file (matrix) | svg |
| 146 | C A T G A G T C G A C T C G T A C G A T G C A T G A C T G C A T C G A T C T A G C A T G T G A C | Mef2b(MADS)/HEK293-Mef2b.V5-ChIP-Seq(GSE67450)/Homer | 1e-2 | -6.296e+00 | 0.0056 | 2549.0 | 13.77% | 4112.4 | 13.04% | motif file (matrix) | svg |
| 147 | C T A G G C A T G A T C C G T A A G T C T C A G G A C T C T A G | CLOCK(bHLH)/Liver-Clock-ChIP-Seq(GSE39860)/Homer | 1e-2 | -6.230e+00 | 0.0059 | 1249.0 | 6.75% | 1963.9 | 6.23% | motif file (matrix) | svg |
| 148 | A G T C C T A G C T A G A G T C G A T C G T A C A G T C C T A G A G T C A G T C A G T C G T A C | Sp2(Zf)/HEK293-Sp2.eGFP-ChIP-Seq(Encode)/Homer | 1e-2 | -6.071e+00 | 0.0069 | 2283.0 | 12.33% | 3676.0 | 11.66% | motif file (matrix) | svg |
| 149 | A T G C A G C T T C A G T G A C T C A G A T G C T G C A A C G T A T C G G A T C A C T G A G T C | NRF1(NRF)/MCF7-NRF1-ChIP-Seq(Unpublished)/Homer | 1e-2 | -5.968e+00 | 0.0076 | 173.0 | 0.93% | 236.3 | 0.75% | motif file (matrix) | svg |
| 150 | T C G A G C A T A T G C C T G A A T G C T A G C A G T C G T A C T C G A A G C T | Srebp1a(bHLH)/HepG2-Srebp1a-ChIP-Seq(GSE31477)/Homer | 1e-2 | -5.950e+00 | 0.0076 | 513.0 | 2.77% | 771.7 | 2.45% | motif file (matrix) | svg |
| 151 | G C T A T C G A C G T A C T A G A G C T G T C A G T C A C G T A A G T C C G T A | FOXA1(Forkhead)/MCF7-FOXA1-ChIP-Seq(GSE26831)/Homer | 1e-2 | -5.826e+00 | 0.0086 | 3266.0 | 17.64% | 5321.5 | 16.88% | motif file (matrix) | svg |
| 152 | T C A G A C G T A G T C T C G A A G T C T C A G G C A T C T A G C T A G A G C T | Usf2(bHLH)/C2C12-Usf2-ChIP-Seq(GSE36030)/Homer | 1e-2 | -5.725e+00 | 0.0094 | 604.0 | 3.26% | 920.9 | 2.92% | motif file (matrix) | svg |
| 153 | T C A G C T A G C T A G A C T G A C T G G A T C A C T G A C T G C T A G C T A G A G T C G A T C | KLF1(Zf)/HUDEP2-KLF1-CutnRun(GSE136251)/Homer | 1e-2 | -5.549e+00 | 0.0112 | 1021.0 | 5.51% | 1601.8 | 5.08% | motif file (matrix) | svg |
| 154 | C G A T A C G T A C G T A C G T C G T A A G C T C A G T C T A G A T C G A C T G | HOXB13(Homeobox)/ProstateTumor-HOXB13-ChIP-Seq(GSE56288)/Homer | 1e-2 | -5.456e+00 | 0.0122 | 3459.0 | 18.68% | 5655.9 | 17.94% | motif file (matrix) | svg |
| 155 | C G T A C T G A C G T A C T A G T C G A C T A G A C T G C G T A C G T A T A C G A G C T A T C G | SpiB(ETS)/OCILY3-SPIB-ChIP-Seq(GSE56857)/Homer | 1e-2 | -5.328e+00 | 0.0138 | 694.0 | 3.75% | 1071.4 | 3.40% | motif file (matrix) | svg |
| 156 | C A T G G T A C C G T A A G T C C T A G A C G T A C T G G T A C A G T C A G C T | bHLHE40(bHLH)/HepG2-BHLHE40-ChIP-Seq(GSE31477)/Homer | 1e-2 | -5.294e+00 | 0.0142 | 730.0 | 3.94% | 1130.9 | 3.59% | motif file (matrix) | svg |
| 157 | A T G C A T G C A T C G T A C G A G C T A G T C G C T A A G T C T C A G G A C T A C T G T C G A | E-box(bHLH)/Promoter/Homer | 1e-2 | -5.224e+00 | 0.0151 | 114.0 | 0.62% | 151.2 | 0.48% | motif file (matrix) | svg |
| 158 | T C A G T G A C G T A C T G C A G T A C C T A G G T A C A T G C A G T C G T C A A G T C G A C T | Klf9(Zf)/GBM-Klf9-ChIP-Seq(GSE62211)/Homer | 1e-2 | -5.223e+00 | 0.0151 | 371.0 | 2.00% | 552.7 | 1.75% | motif file (matrix) | svg |
| 159 | C T G A C T A G T G C A C T G A T C G A A G C T C A T G T C G A A G T C G A C T A C G T A G T C G A T C G A T C G A C T | ZNF528(Zf)/HEK293-ZNF528.GFP-ChIP-Seq(GSE58341)/Homer | 1e-2 | -5.216e+00 | 0.0151 | 21.0 | 0.11% | 19.9 | 0.06% | motif file (matrix) | svg |
| 160 | C T G A A C G T A C G T A C G T A G T C G A C T C G A T C T G A A C T G C G T A C G T A T C G A | STAT5(Stat)/mCD4+-Stat5-ChIP-Seq(GSE12346)/Homer | 1e-2 | -5.205e+00 | 0.0151 | 946.0 | 5.11% | 1484.3 | 4.71% | motif file (matrix) | svg |
| 161 | A G T C C G A T A C T G A T C G T G A C G C T A C A T G A T C G T G A C C G A T A C T G T A G C G T A C G T C A | Tlx?(NR)/NPC-H3K4me1-ChIP-Seq(GSE16256)/Homer | 1e-2 | -5.167e+00 | 0.0156 | 1045.0 | 5.64% | 1647.7 | 5.23% | motif file (matrix) | svg |
| 162 | A C G T C T A G A G C T A C G T A C G T C T G A A G T C G A C T A G C T C G T A | FOXM1(Forkhead)/MCF7-FOXM1-ChIP-Seq(GSE72977)/Homer | 1e-2 | -5.160e+00 | 0.0156 | 3388.0 | 18.30% | 5545.4 | 17.59% | motif file (matrix) | svg |
| 163 | T A C G C T G A C A T G G A T C G T A C G C A T T C A G T A C G A G C T G T C A G A T C G C A T T A C G C G T A C T A G G A T C G A T C C G A T A C T G T C A G | ZNF322(Zf)/HEK293-ZNF322.GFP-ChIP-Seq(GSE58341)/Homer | 1e-2 | -5.157e+00 | 0.0156 | 613.0 | 3.31% | 943.0 | 2.99% | motif file (matrix) | svg |
| 164 | C T A G C A T G T G C A A C G T A G T C C G T A C A T G T C A G A C G T A C G T G C T A A G T C | Six1(Homeobox)/Myoblast-Six1-ChIP-Chip(GSE20150)/Homer | 1e-2 | -5.130e+00 | 0.0159 | 614.0 | 3.32% | 944.5 | 3.00% | motif file (matrix) | svg |
| 165 | C T G A T A C G G C A T A G C T A G C T A G T C T C G A A C T G C A G T A G C T A G C T G A T C | IRF3(IRF)/BMDM-Irf3-ChIP-Seq(GSE67343)/Homer | 1e-2 | -5.100e+00 | 0.0163 | 1026.0 | 5.54% | 1617.8 | 5.13% | motif file (matrix) | svg |
| 166 | C A T G A C G T A G T C G A T C G A T C G A T C G C T A C T A G C T A G C T A G T C A G T C G A | EBF1(EBF)/Near-E2A-ChIP-Seq(GSE21512)/Homer | 1e-2 | -4.838e+00 | 0.0210 | 2115.0 | 11.42% | 3426.4 | 10.87% | motif file (matrix) | svg |
| 167 | T C G A C A T G C A T G T A C G G T A C T C G A A G T C C A G T C T G A C G T A A G T C G A C T | ZNF264(Zf)/HEK293-ZNF264.GFP-ChIP-Seq(GSE58341)/Homer | 1e-2 | -4.787e+00 | 0.0220 | 1530.0 | 8.26% | 2455.1 | 7.79% | motif file (matrix) | svg |
| 168 | C T G A A G C T A C G T A C G T A G T C G A C T G A C T C T G A C T G A C T A G C G T A C G T A | STAT6(Stat)/CD4-Stat6-ChIP-Seq(GSE22104)/Homer | 1e-2 | -4.748e+00 | 0.0227 | 1815.0 | 9.80% | 2929.4 | 9.29% | motif file (matrix) | svg |
| 169 | T G C A G C A T A G C T G C A T A G T C A G T C A G T C C T G A A C T G T C G A T C G A C A G T A T C G A G T C G A T C | ZNF143\|STAF(Zf)/CUTLL-ZNF143-ChIP-Seq(GSE29600)/Homer | 1e-2 | -4.738e+00 | 0.0228 | 594.0 | 3.21% | 917.7 | 2.91% | motif file (matrix) | svg |
| 170 | A C T G A C G T C A T G A T C G A T C G T G A C A C T G A T C G A T C G T G C A C T G A C G T A | E2F3(E2F)/MEF-E2F3-ChIP-Seq(GSE71376)/Homer | 1e-2 | -4.707e+00 | 0.0234 | 1035.0 | 5.59% | 1639.4 | 5.20% | motif file (matrix) | svg |
| 171 | T A C G T C A G G A T C G T A C T C G A G A C T G C T A G C T A G C T A C G T A G A T C G T C A | CDX4(Homeobox)/ZebrafishEmbryos-Cdx4.Myc-ChIP-Seq(GSE48254)/Homer | 1e-2 | -4.676e+00 | 0.0240 | 3005.0 | 16.23% | 4918.4 | 15.60% | motif file (matrix) | svg |
| 172 | T A G C G A T C G T A C C T G A G C A T C T G A C G T A T G C A C G T A G A T C | Hoxa13(Homeobox)/ChickenMSG-Hoxa13.Flag-ChIP-Seq(GSE86088)/Homer | 1e-2 | -4.674e+00 | 0.0240 | 7966.0 | 43.02% | 13296.0 | 42.16% | motif file (matrix) | svg |
| 173 | T G A C C T G A C T A G T C G A C T G A A T G C C G T A A C T G G C A T G T A C G C A T A T C G G C A T A G C T G A T C | PR(NR)/T47D-PR-ChIP-Seq(GSE31130)/Homer | 1e-2 | -4.649e+00 | 0.0243 | 6793.0 | 36.69% | 11307.0 | 35.86% | motif file (matrix) | svg |
| 174 | A T G C A G T C G T A C A G C T T C G A C T A G G A T C C T G A G T C A A G T C G C T A T C A G | Rfx5(HTH)/GM12878-Rfx5-ChIP-Seq(GSE31477)/Homer | 1e-2 | -4.627e+00 | 0.0247 | 736.0 | 3.97% | 1150.6 | 3.65% | motif file (matrix) | svg |
| 175 | T A C G T C A G T G C A A G C T T G A C A G C T A G T C A C T G G A T C A C T G T C G A A C T G C T G A C T G A A T G C | ZBTB33(Zf)/GM12878-ZBTB33-ChIP-Seq(GSE32465)/Homer | 1e-2 | -4.609e+00 | 0.0250 | 46.0 | 0.25% | 54.5 | 0.17% | motif file (matrix) | svg |
